# Effects of Ghrelin on Triglyceride Accumulation and Glucose Uptake in Primary Cultured Rat Myoblasts under Palmitic Acid-Induced High Fat Conditions

**DOI:** 10.1155/2015/635863

**Published:** 2015-12-02

**Authors:** Lingling Han, Jia Li, Ying Chen, Wei Wang, Dan Zhang, Guoliang Liu

**Affiliations:** ^1^Department of Endocrinology, The First Hospital of China Medical University, Shenyang, Liaoning 110001, China; ^2^Department of Endocrinology, The Fourth Hospital of China Medical University, Shenyang, Liaoning 110032, China; ^3^Shenyang 93303 Military Hospital, Shenyang, Liaoning 110011, China

## Abstract

This study aimed to study the effects of acylated ghrelin on glucose and triglyceride metabolism in rat myoblasts under palmitic acid- (PA-) induced high fat conditions. Rat myoblasts were treated with 0, 10^−11^, 10^−9^, or 10^−7^ M acylated ghrelin and 0.3 mM PA for 12 h. Triglyceride accumulation was determined by Oil-Red-O staining and the glycerol phosphate dehydrogenase-peroxidase enzymatic method, and glucose uptake was determined by isotope tracer. The glucose transporter 4 (GLUT4), AMP-activated protein kinase (AMPK), acetyl-CoA carboxylase (ACC), and uncoupling protein 3 (UCP3) were assessed by RT-PCR and western blot. Compared to 0.3 mM PA, ghrelin at 10^−9^ and 10^−7^ M reduced triglyceride content (5.855 ± 0.352 versus 5.030 ± 0.129 and 4.158 ± 0.254 mM, *P* < 0.05) and prevented PA-induced reduction of glucose uptake (1.717 ± 0.264 versus 2.233 ± 0.333 and 2.333 ± 0.273 10^−2^ pmol/g/min, *P* < 0.05). The relative protein expression of p-AMPK*α*/AMPK*α*, UCP3, and p-ACC under 0.3 mM PA was significantly reduced compared to controls (all *P* < 0.05), but those in the 10^−9^ and 10^−7^ M ghrelin groups were significantly protected from 0.3 mM PA (all *P* < 0.05). In conclusion, acylated ghrelin reduced PA-induced triglyceride accumulation and prevented the PA-induced decrease in glucose uptake in rat myoblasts. These effects may involve fatty acid oxidation.

## 1. Introduction

The probability of any animal surviving famine is based on its energy stores (mainly as fat) [1]. Adipocytes uptake and store free fatty acids (FFA) as triglycerides (TG) [2]. In periods of low energy intake or fasting, adipocytes release FFA and glycerol to be used as fuel. High FFA levels reduce the insulin-stimulated uptake and use of glucose by tissues, preserving the glucose for vital tissues such as the central nervous system [3]. A sustained FFA release for more than a few hours can induce insulin resistance, which could contribute to the development of type 2 diabetes (T2DM) if it is sustained over long periods of time [4]. Studies have shown that high FFA levels are an important pathogenic factor for obesity-induced insulin resistance [4]. In situations of continuous energy excess, as experienced by many people in western countries, FFA-induced insulin resistance is pointless because there is no need for glucose preservation [4]. Another mechanism leading to insulin resistance is through increased oxidative stress by high FFA levels, leading to activation of the protein kinase C (PKC) and nuclear factor-*κ*B (NF-*κ*B) pathways [4, 5].

Skeletal muscles uptake and use a large part of available glucose and TG [6]. Insulin resistance and islet *β*-cell failure are the two main features of T2DM. Increased FFA levels and TG accumulation in nonadipose tissues are closely related to these two features [7]. Studies in nonobese and nondiabetic offspring of diabetic patients showed that increased levels of intracellular fat in muscle cells were an early abnormality associated with insulin resistance [4]. Such abnormalities may result in abnormal glucose uptake [8].

In the basal state, skeletal muscle consumes about 20% of total glucose, compared to 75–95% under glucose stimulation [9]. Transmembrane transportation of glucose limits the rate of blood glucose uptake in skeletal muscles. The glucose transporter 4 (GLUT4) is the main glucose transportation protein in skeletal muscles [10]. The amount of GLUT4 proteins on the cell membrane is directly correlated to the amount of blood glucose that can be transported across the cell membrane. The decreased ability of skeletal muscle to uptake blood glucose might be associated with impairments of the GLUT4 mechanisms [11]. AMP-activated protein kinase (AMPK) is the major signaling factor for glucose transportation in skeletal muscles and is the cause of GLUT4 transposition to the cell membrane [12]. AMPK can inactivate phosphorylated acetyl-CoA carboxylase (ACC), thus reducing malonyl-CoA and TG synthesis and increasing fatty acid oxidation. In this way, fatty toxicity is reduced and the sensitivity to insulin is increased [13, 14]. After AMPK activation, the uncoupling protein 3 (UCP3) can transport fatty acids. Bezaire et al. [15] have shown that when the expression of UCP3 in rat skeletal muscle was increased, the intracellular fat content was decreased, indicating increased fatty acid oxidation.

Many hormones play roles in the metabolism of lipids and glucose [16–18]. A number of hormones from the digestive system (like insulin, glucagon, and ghrelin) [17, 18] and from the adipose tissue (like leptin and adiponectin) [19] play direct roles in lipid and glucose stores and use. Among them, ghrelin is a peptide hormone produced by ghrelinergic cells in the gastrointestinal tract [20]. It is an endogenous ligand of the growth hormone secretagogue receptor (GHS-R) and binds to different GHS-R subtypes in peripheral tissues to regulate physiological functions such as energy metabolism, gastrointestinal function, tumor cell proliferation, cardiovascular function, and hormone release [20]. Recent studies have revealed an association between ghrelin and insulin resistance and the regulation of insulin secretion [21, 22]. Studies have shown that plasma ghrelin levels in obese patients with T2DM were significantly lower than in nonobese patients and that ghrelin levels were associated with the amount of visceral fat, hyperproinsulinemia, and insulin resistance [23]. After intravenous injection of ghrelin, Barazzoni et al. observed that ghrelin facilitates glucose uptake by skeletal muscles and increases their insulin sensitivity [21, 24].

In this study, a model of fatty degeneration was established by treating primary cultured rat myoblasts with palmitic acid (PA). This study aimed to investigate the effects of acylated ghrelin on glucose uptake and TG accumulation and to examine the underlying molecular mechanisms.

## 2. Materials and Methods

### 2.1. Animals

Four newborn male Wistar rats (3 days old; body weight: 5.8–6.2 g) were obtained from the Laboratory Animal Center of China Medical University. All four rats were born from and breastfed by the same Wistar female. The female was housed at 20–25°C and 50 ± 5% humidity with ad libitum access to food and water and under a 12 : 12 h light/dark cycle. The composition of the diet provided to the female was 28.6% protein, 9.6% fat, and 61.8% carbohydrates. Newborn rats were used for myoblast extraction 3 days after being born. All procedures and animal experiments were approved by the Animal Care and Use Committee of China Medical University.

### 2.2. Culture and Identification of Rat Myoblasts

**Figure 1:**
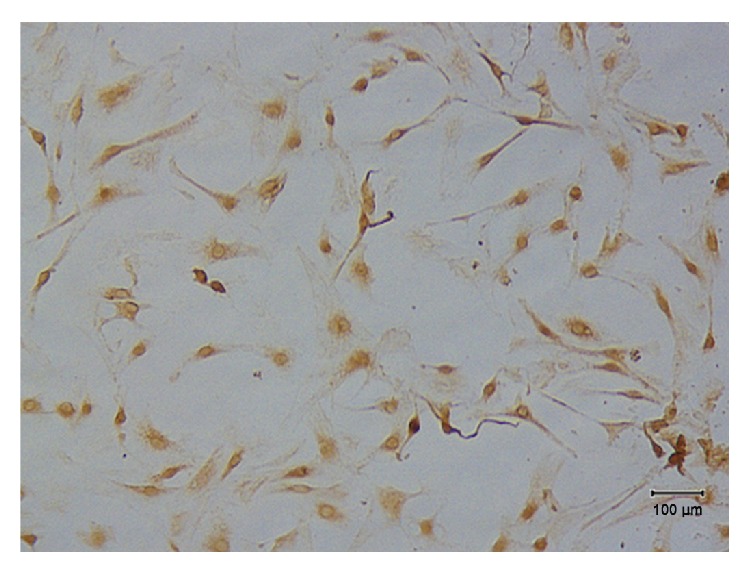
Identification of rat myoblasts. *α*-actin was determined by immunohistochemistry (scale bar: 100 *μ*m). Cytoplasm of rat myoblasts appears clay-white.

The lower limb and abdominal muscles were separated under aseptic conditions and cut into 1 mm^3^ pieces. Samples were digested with type I collagenase (Sigma, St. Louis, MI, USA) for 40 min and 0.25% trypsin for 30 min, filtered through a 200 *µ*m mesh filter, and centrifuged at 1000 rpm for 10 min. The supernatants were discarded and Dulbecco's Modified Eagle Medium (DMEM; Hyclone, Thermo Fisher Scientific, Waltham, MA, USA) containing 10% fetal bovine serum was added. Cells were inoculated into a 6-well plate at 10^6^ cells/mL and cultured at 37°C in an atmosphere of 5% CO_2_. After 24 h, cells were digested with pancreatin and reinoculated into a 75 cm^2^ culture flask at 2 × 10^6^ cells/mL. The medium was replaced every 2-3 days. When the cells reached 80–90% confluence, they were released by digestion with 0.25% trypsin and passaged. For purification, the differential adhesion method was used. The cultured cells were inoculated into a 24-well plate. Rabbit anti-*α*-actin antibodies were used for identification by immunohistochemistry (Figure 1).

### 2.3. Rat Myoblast Treatments

When the primary culture cells reached 80–90% confluence, cells in the control group were transferred to DMEM containing 0.5% bovine serum albumin (BSA; Sigma, St. Louis, MI, USA) for 12 h. Cells in the high fat group were cultured with 0.3 mM PA (Sigma) (DMEM containing 0.5% BSA) for 12 h [25]. In the three intervention groups, acylated ghrelin (Phoenix Pharmaceuticals, Inc., Burlingame, CA, USA) was added at 10^−11^ M, 10^−9^ M, or 10^−7^ M along with 0.3 mM PA for 12 h. Ghrelin was reconstituted in ddH_2_O to create the stock solution and further diluted with DMEM containing 0.5% BSA before being added to the wells.

### 2.4. Cell Proliferation Assay

Proliferation of myoblasts was measured using a MTT assay [26]. In brief, MTT (5 mg/mL; 20 *μ*L; Roche, Indianapolis, IN, USA) was added to each well and incubated for 4 h. After careful removal of the culture medium, DMSO (150 *μ*L) was added. The absorbance of each well was measured at 490 nm with a microplate spectrophotometer (Bio-tek, ELx800, USA).

### 2.5. Lipid Metabolism

TG accumulation after fatty degeneration of myoblasts was measured by Oil-Red-O staining (Solarbio Science & Technology Co., Ltd., Beijing, China) [27]. TG content was measured by the glycerol phosphate dehydrogenase-peroxidase (GPO-POD) enzyme method (Applygen Technologies Inc., Beijing, China) [28].

### 2.6. Glucose Metabolism

The uptake of 2-deoxy-D-[^3^H] glucose (China Institute of Atomic Energy) was measured by the isotope tracer technique (liquid scintillation counter (PerkinElmer MicroBeta, USA)) [29].

### 2.7. Real-Time Polymerase Chain Reaction

Real-time polymerase chain reaction (RT-PCR) was performed to measure the mRNA expression of GLUT4, AMPK, and UCP3 [30]. The primers and RT-PCR conditions are listed in Table 1. The amplified products were analyzed by 1.5% agarose gel electrophoresis. Gray-scale analysis was performed using the ChemiImager 5500 gel imaging system (Alpha Innotech Corporation, USA) with FluorChem v2.0 software (Alpha Innotech Corporation, USA). The relative expression rate of target mRNA was represented as the ratio of the gray-scale value between the mRNA under investigation and the reference gene, GAPDH.

### 2.8. Western Blot

Western blot was performed to measure the protein levels of GLUT4, AMPK*α*, UCP3, and p-ACC [31] using rabbit anti-rat polyclonal antibody against AMPK*α*, p-AMPK*α*, GLUT4, p-ACC, and UCP3 (all from Cell Signaling, USA). Following overnight incubation with the primary antibody, blots were incubated with horseradish peroxidase- (HRP-) conjugated goat anti-rabbit secondary antibodies (Cell Signaling, USA). The scanned blots were subjected to gray-scale analysis. Relative expression of target proteins was expressed as a gray-scale value ratio between proteins and internal reference *β*-actin.

### 2.9. Statistical Analysis

Statistical analysis was performed using SPSS 13.0 (SPSS Inc., Chicago, IL, USA). Data are expressed as mean ± standard deviation (SD) of three independent experiments performed in triplicate. Statistical significance was evaluated by one-way analysis of variance (ANOVA) with the Student-Newman-Keuls (SNK) post hoc test. *P* values < 0.05 were considered significantly different.

## 3. Results

### 3.1. Effects of Acylated Ghrelin on PA-Induced TG Accumulation in Primary Cultured Rat Myoblasts

**Figure 2:**
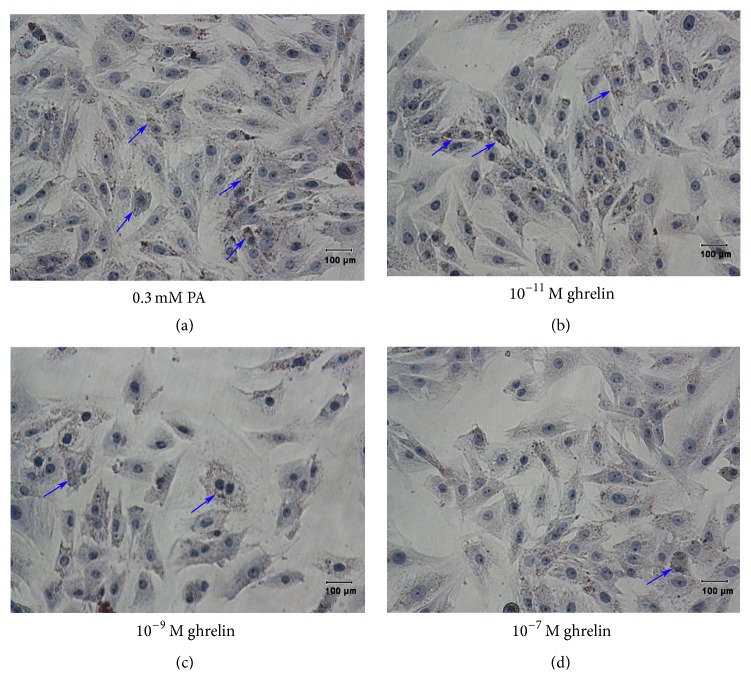
Effects of acylated ghrelin on palmitic acid- (PA-) induced triglyceride (TG) accumulation in primary cultured rat myoblasts. Rat myoblasts in the high fat group were cultured with 0.3 mM PA (containing 0.5% BSA) for 12 h (a). In the three intervention groups, acylated ghrelin at 10^−11^ M (b), 10^−9^ M (c), or 10^−7^ M (d) was added under high fat conditions for 12 h. TG accumulation (blue arrows) was determined by Oil-Red-O staining (scale bar: 100 *μ*m).

No TG deposits were observed in the control cells (data not shown). Cells with fatty degeneration were observed after being cultured for 12 h with 0.3 mM PA. Many fat droplets were present in the cytoplasm (Figure 2, blue arrows). There were less fat droplets in the ghrelin intervention groups.

**Figure 3:**
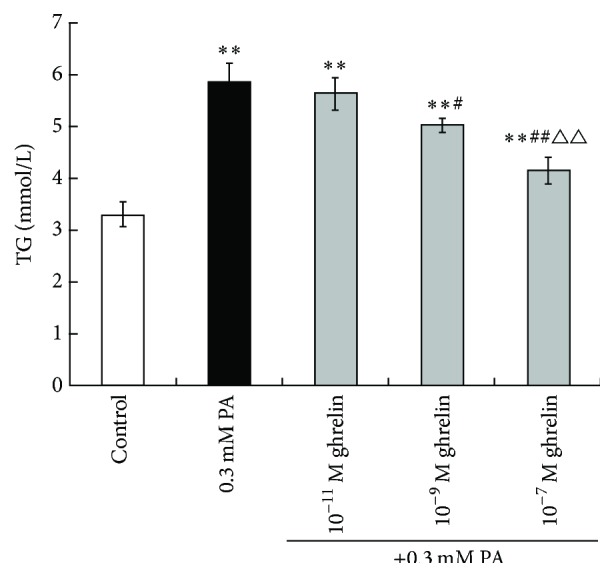
Effects of acylated ghrelin on PA-induced TG accumulation in primary cultured rat myoblasts. TG content was measured by the glycerol phosphate dehydrogenase-peroxidase enzymatic method. Data are shown as mean ± standard deviation (SD). ^*∗∗*^ *P* < 0.01 versus controls; ^#^ *P* < 0.05, ^##^ *P* < 0.01 versus the 0.3 mM PA group; ^△△^ *P* < 0.01 versus the 0.3 mM PA + 10^−11^ M acylated ghrelin group.

As shown in Figure 3, 0.3 mM PA treatment increased the TG content of rat myoblasts compared to controls (5.855 ± 0.352 versus 3.303 ± 0.235 mM, *P* < 0.01). Myoblasts treated with 0.3 mM PA and 10^−11^ M acylated ghrelin showed TG contents that were elevated compared to controls (5.638 ± 0.308 mM, *P* < 0.01 versus controls), but comparable to that of 0.3 mM PA-treated cells (*P* > 0.05). Myoblasts treated with 0.3 mM PA and 10^−9^ M acylated ghrelin showed that TG content was elevated compared to controls but reduced compared to 0.3 mM PA (5.030 ± 0.129 mM, *P* < 0.01 versus controls, *P* < 0.05 versus 0.3 mM PA). Myoblasts treated with 0.3 mM PA and 10^−7^ M acylated ghrelin showed TG contents that were elevated compared to controls but reduced compared to 0.3 mM PA and 0.3 mM PA and 10^−11^ M acylated ghrelin (4.158 ± 0.254 mM, *P* < 0.01 versus controls, *P* < 0.01 versus 0.3 mM PA, and *P* < 0.01 versus 0.3 mM PA and 10^−11^ M acylated ghrelin) (Figure 3). These results suggest that acylated ghrelin protected the cells from PA-induced TG accumulation in rat myoblasts under high fat conditions, in a dose-dependent manner.

### 3.2. Effects of Acylated Ghrelin on Glucose Uptake in Primary Cultured Rat Myoblasts under High Fat Conditions

**Figure 4:**
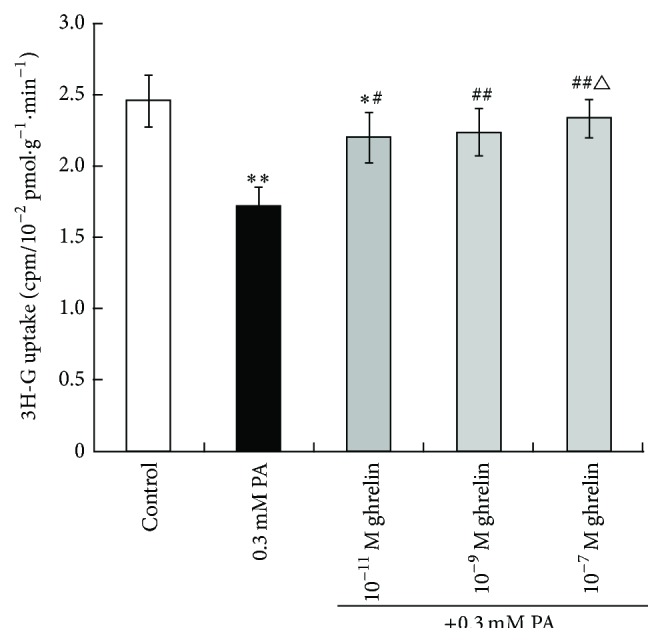
Effects of acylated ghrelin on glucose uptake in primary cultured rat myoblasts under high fat conditions. The uptake of 2-deoxy-D-[^3^H] glucose was measured by the isotope tracer technique. Data are shown as mean ± SD. ^*∗*^ *P* < 0.05, ^*∗∗*^ *P* < 0.01 versus controls; ^#^ *P* < 0.05, ^##^ *P* < 0.01 versus the 0.3 mM PA group; ^△^ *P* < 0.05 versus the 0.3 mM PA + 10^−11^ M acylated ghrelin group.

As shown in Figure 4, glucose uptake was reduced in myoblasts treated with 0.3 mM PA or 10^−11^ M acylated ghrelin compared to controls (1.717 ± 0.264 and 2.200 ± 0.352 versus 2.350 ± 0.362 cpm/10^−2^ pmol·g^−1^·min^−1^; *P* < 0.01 and *P* < 0.05, resp.). Compared to the 0.3 mM PA group, cells in the 10^−9^ M acylated ghrelin group had significantly higher glucose uptake (2.233 ± 0.333 cpm/10^−2^ pmol·g^−1^·min^−1^, *P* < 0.01); and cells in the 10^−7^ M acylated ghrelin group had significantly higher glucose uptake (2.333 ± 0.273 cpm/10^−2^ pmol·g^−1^·min^−1^, *P* < 0.01 versus 0.3 mM PA, *P* < 0.05 versus 10^−11^ M acylated ghrelin). These results suggest that acylated ghrelin prevented the PA-induced decrease in glucose uptake in rat myoblasts under high fat conditions, in a dose-dependent manner.

### 3.3. Effects of Acylated Ghrelin on GLUT4, AMPK, UCP3, and p-ACC mRNA and Protein Expression in Rat Myoblasts under High Fat Condition

**Figure 5:**
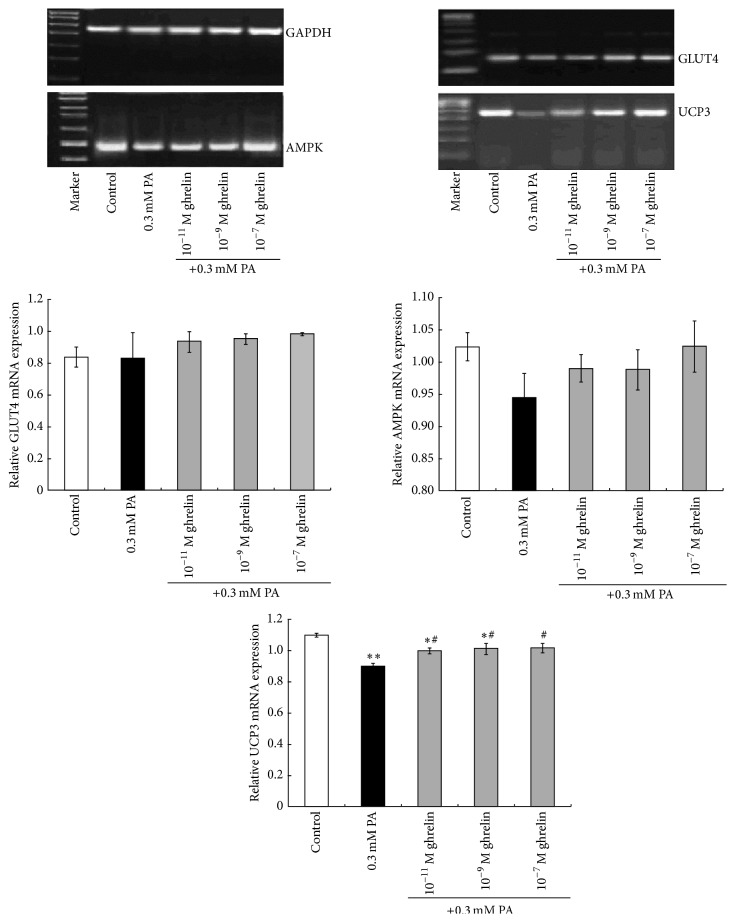
Effects of acylated ghrelin on the mRNA expression of genes involved in lipid and glucose metabolism. mRNA expression of the glucose transporter 4 (GLUT4), AMP-activated protein kinase (AMPK), and uncoupling protein 3 (UCP3) in primary cultured rat myoblasts under high fat conditions was determined by real-time polymerase chain reaction. Glyceraldehyde 3-phosphate dehydrogenase (GAPDH) was used as internal reference. Data are shown as mean ± SD. ^*∗*^ *P* < 0.05, ^*∗∗*^ *P* < 0.01 versus controls; ^#^ *P* < 0.05 versus the 0.3 mM PA group.

**Figure 6:**
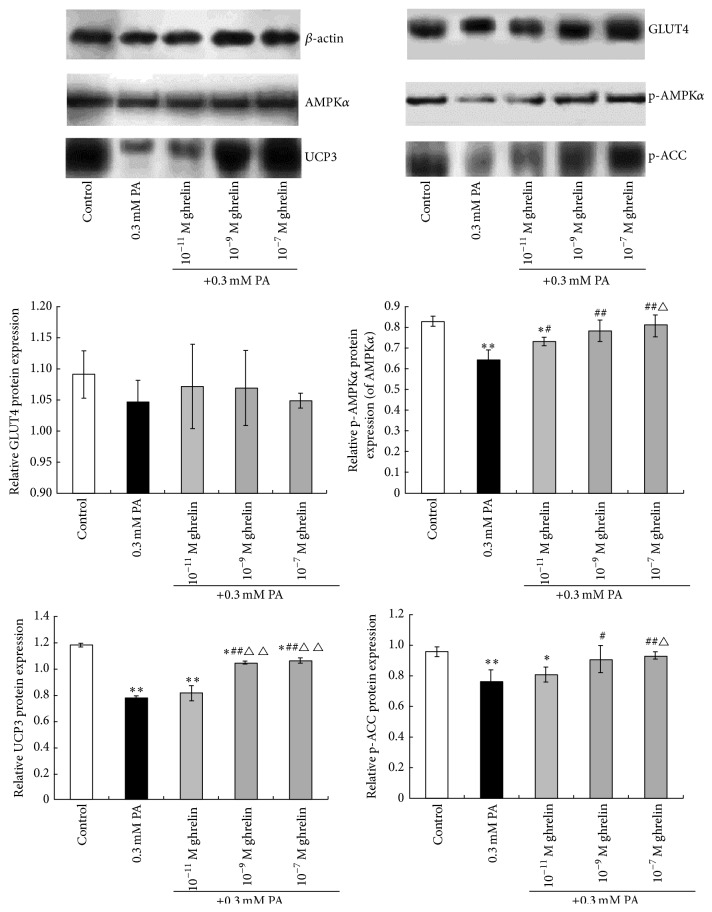
Effects of acylated ghrelin on the protein expression of proteins involved in lipid and glucose metabolism. Protein expression of glucose transporter 4 (GLUT4), AMP-activated protein kinase *α* (AMPK*α*), phosphorylated AMPK*α* (p-AMPK*α*), uncoupling protein 3 (UCP3), and phosphorylated acetyl-CoA carboxylase (p-ACC) in primary cultured rat myoblasts under high fat conditions was determined by western blot. *β*-actin was used as internal reference. Data are shown as mean ± SD. ^*∗*^ *P* < 0.05, ^*∗∗*^ *P* < 0.01 versus controls; ^#^ *P* < 0.05, ^##^ *P* < 0.01 versus the 0.3 mM PA group; ^△^ *P* < 0.05, ^△△^ *P* < 0.01 versus the 0.3 mM PA + 10^−11^ M acylated ghrelin group.

As shown in Figures 5 and 6, there were no differences in the relative expression of GLUT4 mRNA and protein between the high fat, control, and acylated ghrelin intervention groups (all *P* > 0.05).

As shown in Figure 5, there were no significant differences in the relative mRNA expression of AMPK between the control, high fat, and acylated ghrelin intervention groups. The p-AMPK*α*/AMPK*α* ratio in the 0.3 mM PA and the 10^−11^ M acylated ghrelin groups was significantly lower than in controls (*P* < 0.05 and *P* < 0.01, resp.), while the differences between the 10^−7^ M and 10^−9^ M acylated ghrelin groups were not statistically significant. However, the p-AMPK*α*/AMPK*α* ratio was significantly higher in the 10^−7^ M and 10^−9^ M acylated ghrelin groups compared to the 0.3 mM PA group (both *P* < 0.01). The p-AMPK*α*/AMPK*α* ratio in the 10^−7^ M acylated ghrelin group was significantly higher than in the 10^−11^ M acylated ghrelin group (*P* < 0.05) (Figure 6).

As shown in Figures 5 and 6, the relative expression of UCP3 mRNA and protein in the 0.3 mM PA group was significantly lower compared to controls (both *P* < 0.01), but expression of UCP3 mRNA and protein in the three acylated ghrelin intervention groups was significantly higher compared to the 0.3 mM PA group (all *P* < 0.05, except protein expression at 10^−11^ M acylated ghrelin). There were no significant differences in the relative UCP3 mRNA expression between all acylated ghrelin treatment groups.

As shown in Figure 6, compared to controls, the relative protein content of p-ACC in the 0.3 mM PA and 10^−11^ M acylated ghrelin groups was significantly lower (*P* < 0.05 and *P* < 0.01, resp.). Compared to the 0.3 mM PA group, the relative protein content of p-ACC in the 10^−9^ M and 10^−7^ M acylated ghrelin groups was significantly higher (*P* < 0.05 and *P* < 0.01, resp.). The relative protein content of p-ACC in the 10^−7^ M acylated ghrelin group was significantly higher than in the 10^−11^ M acylated ghrelin group (*P* < 0.05). No significant differences were found in the relative protein content of p-ACC between the 10^−7^ M and 10^−9^ M acylated ghrelin groups.

## 4. Discussion

The aim of this study was to examine the effects of acylated ghrelin on glucose and TG metabolism in primary cultured rat myoblasts under PA-induced high fat conditions. Compared to 0.3 mM PA, acylated ghrelin at 10^−9^ and 10^−7^ M resulted in lower TG content and higher glucose uptake. There were no differences in GLUT4 mRNA and protein. The relative protein expression of p-AMPK*α*/AMPK*α*, UCP3, and p-ACC in the 0.3 mM PA group was significantly lower compared to controls (all *P* < 0.05), but those in the 10^−9^ and 10^−7^ M acylated ghrelin groups were significantly higher compared to the 0.3 mM PA group (all *P* < 0.05). These results suggest that acylated ghrelin prevented the PA-induced TG accumulation and protected the cells against PA-induced decrease in glucose uptake in cultured rat myoblasts under PA-induced high fat conditions. These effects may involve fatty acid oxidation.

The role of ghrelin in insulin sensitivity is complex. Indeed, previous studies demonstrated that obesity was associated with an imbalance in the levels of unacylated and acylated ghrelin and that a relative excess of acylated ghrelin could be associated with insulin resistance in human [21]. On the other hand, another study has shown that the concomitant administration of acylated and unacylated ghrelin to obese nondiabetic patients increased their insulin sensitivity [22]. Zhang et al. have shown that ghrelin inhibited fat formation through a receptor regulation-based stimulation of cell proliferation [32]. Barazzoni et al. have shown that ghrelin injections in rats improved insulin sensitivity in the skeletal muscles but not in the liver [33]. Accordingly, using* in vitro* culture of rat myoblasts, this study confirmed the hypothesis that acylated ghrelin could promote glucose uptake. In order to construct a model of fatty degeneration induced by high fat conditions, rat myoblasts were treated with PA to induce TG accumulation. Decreased glucose uptake was then observed, as supported by previously studies [25]. Acylated ghrelin reduced TG accumulation in a dose-dependent manner in rat myoblasts with PA-induced fatty degeneration. Moreover, acylated ghrelin improved glucose uptake in these cells. These results strongly suggest that acylated ghrelin counteracted the effects of the high fat environment. However, no formal dose-response experiment was performed in this study, and the lack of effect of 10^−11^ M acylated ghrelin might be because the concentration was simply too low to exert any detectable effect.

Compared to controls, the relative mRNA and protein expression of GLUT4 did not differ significantly between the high fat and acylated ghrelin treatment groups. A previous study has shown that ghrelin increased the mRNA levels of GLUT4 [34]. However, other studies observed that no significant changes in GLUT4 expression were observed in myoblasts under basal conditions or under insulin stimulation [35]. The relative mRNA and protein expression of GLUT4 in myoblasts are similar in normal, obese, and diabetic subjects [36]. A previous study has shown that ghrelin regulates the adipocyte metabolism toward fat accumulation via increased glucose and TG uptake [37]. Thus, based on previous reports, the inhibition of glucose uptake under high fat conditions might be related to the activity of GLUT4 and its intramembrane and extramembrane distribution, not on its expression level.

Compared to the high fat group, the relative expression of p-AMPK*α*/AMPK*α* in the acylated ghrelin treatment groups was significantly increased in a dose-dependent manner. Previous studies have shown that ghrelin secreted by the gastrointestinal tract inhibited AMPK activity in the liver and white adipose tissue, thus increasing glucose and TG synthesis [38, 39]. However, this study examined myoblasts and showed that ghrelin activated AMPK in myoblasts, resulting in protection from PA-induced TG accumulation and from PA-induced decrease in glucose uptake. It could therefore be inferred that, by facilitating AMPK phosphorylation, ghrelin results in higher glucose uptake and fat metabolism in rat myoblasts. With increasing cytoplasmic FFA concentrations in muscle cells, catabolism of long-chain fatty acids generates excessive ceramides (a known inducer of insulin resistance), which first inhibit glucose transport induced by insulin activation and then inhibit both the function and expression of AMPK [40]. These effects result in the reduced function and expression of UCP3 and p-ACC, followed by lowered muscle glycogen and glucose oxidation, which ultimately lead to insulin resistance [41]. Therefore, it may be hypothesized that acylated ghrelin regulates glucose and TG metabolism in myoblasts via the AMPK pathway.

In this study, the relative mRNA and protein expression of UCP3 and p-ACC in the high fat group were significantly lower than in controls, which is supported by previous studies [42]. Bezaire et al. [15] showed that when the expression of UCP3 in rat skeletal muscle was increased by 2.3-fold over the physiological level, the expression of key fatty acid metabolism enzymes was upregulated. The decreased intracellular fat in muscle cells indicated increased fatty acid oxidation.

Supporting the role of UCP3 in diabetes, drugs for diabetes treatment can restore the expression of UCP3 mRNA in skeletal muscle of patients with T2DM and impaired glucose tolerance [21, 22]. By inactivating ACC through phosphorylation, AMPK causes a reduction in malonyl-CoA and an increase in fatty acid oxidation. At the same time, TG synthesis and lipotoxicity are reduced and insulin sensitivity is increased [13, 14].

The results of this present study show that after acylated ghrelin treatment, the mRNA and protein expression of UCP3 were increased in a dose-dependent manner. The relative protein content of p-ACC was also increased in a similar manner. Therefore, it may be hypothesized that acylated ghrelin upregulated the expression of UCP3 and p-ACC, increased fatty acid oxidation, and reduced TG synthesis. Consequently, in rat myoblasts, lipotoxicity was reduced while glucose transport was increased. Recent studies have shown that ghrelin can increase mitochondrial function and fat metabolism in nonfat tissues (liver and skeletal muscles), thus reducing TG accumulation in skeletal muscle cells [24]. This phenomenon might be associated with increased insulin sensitivity and decreased insulin resistance in skeletal muscles. It could therefore be inferred that by increasing mitochondrial function and fat metabolism in rat myoblasts, ghrelin increased lipid oxidation, decreased TG deposition in rat skeletal muscle cells, and increased glucose transport in skeletal muscles. The exact mechanisms will have to be explored in future studies.

## 5. Conclusions

The present study strongly suggests that acylated ghrelin reduced TG accumulation and promoted glucose uptake in cultured rat myoblasts under PA-induced high fat conditions, which may be involved in the AMPK pathway as well as promoting UCP3 activation and ACC phosphorylation, thus promoting fatty acid oxidation.

## Figures and Tables

**Table 1:** Primers and PCR conditions used for RT-PCR.

Gene	Primers (5′-3′)	Gene location	Product length (bp)	Annealing temperature	Cycles
GLUT4	Forward: GGG CTG TGA GTG AGT GCT TTC Reverse: CAG CGA GGC AAG GCT AGA	chr10	151	50°C	35

AMPK	Forward: TCA GGC ACC CTC ATA TAA TC Reverse: TGA CAA TAG TCC ACA CCA GA	chr2	181	60°C	35

UCP3	Forward: ACG GAT GTG GTG AAG GTC CG Reverse: TAC AAA CAT CAT CAC GTT CC	chr1	465	55°C	35

GAPDH	Forward: ATG GTG AAG GTC GGT GTG AAC Reverse: GCT GAC AAT CTT GAG GGA GT	Multiple	437	52°C	35

GLUT4: glucose transporter 4; AMPK: AMP-activated protein kinase; UCP3: uncoupling protein 3; GAPDH: glyceraldehyde 3-phosphate dehydrogenase.
